# MicroRNA-199b Downregulation Confers Resistance to 5-Fluorouracil Treatment and Predicts Poor Outcome and Response to Neoadjuvant Chemoradiotherapy in Locally Advanced Rectal Cancer Patients

**DOI:** 10.3390/cancers12061655

**Published:** 2020-06-22

**Authors:** Ion Cristóbal, Jaime Rubio, Andrea Santos, Blanca Torrejón, Cristina Caramés, Laura Imedio, Sofía Mariblanca, Melani Luque, Marta Sanz-Alvarez, Sandra Zazo, Juan Madoz-Gúrpide, Federico Rojo, Jesús García-Foncillas

**Affiliations:** 1 Cancer Unit for Research on Novel Therapeutic Targets, Oncohealth Institute, IIS- Fundación Jiménez Díaz-UAM, E-28040 Madrid, Spain; jaime.rubiop@quironsalud.es (J.R.); andreasantos.asc@gmail.com (A.S.); blanca.torrejonmoreno@gmail.com (B.T.); ccarames@fjd.es (C.C.); lauraimedio@gmail.com (L.I.); sofiamariblanca18@gmail.com (S.M.); 2 Translational Oncology Division, Oncohealth Institute, IIS-Fundación Jiménez Díaz-UAM, E-28040 Madrid, Spain; 3 Medical Oncology Department, University Hospital “Fundación Jiménez Díaz”, UAM, E-28040 Madrid, Spain; 4 Pathology Department, IIS-Fundación Jiménez Díaz-UAM, E-28040 Madrid, Spain; melani.luque@quironsalud.es (M.L.); marta.sanza@quironsalud.es (M.S.-A.); Szazo@fjd.es (S.Z.); jmadoz@fjd.es (J.M.-G.); frojo@fjd.es (F.R.)

**Keywords:** MiR-199b, SET, locally advanced rectal cancer, prognosis, pathological response

## Abstract

Neoadjuvant 5-fluorouracil (5-FU)-based chemoradiotherapy followed by mesorectal excision is the current standard treatment in locally advanced rectal cancer (LARC) and the lack of complete response represents a major problem that compromises long-term patient survival. However, there is a lack of robust established markers predictive of response to this preoperative treatment available in the clinical routine. The tumor suppressor microRNA (miR)-199b directly targets the PP2A inhibitor SET, which has been involved in 5-FU resistance, and its downregulation has been found to correlate with poor outcome in metastatic colorectal cancer. Here, we studied the functional effects of miR-199b on 5-FU sensitivity after its ectopic modulation, and its expression was quantified by real-time-PCR in a cohort of 110 LARC patients to evaluate its potential clinical significance. Interestingly, our findings demonstrate that miR-199b enhances the sensitivity of colorectal cancer cells to 5-FU in a SET-dependent manner, and that both miR-199b overexpression and SET inhibition are able to overcome resistance to this drug using an acquired 5-FU-resistant model. MiR-199b was found downregulated in 26.4% of cases and was associated with positive lymph node levels after chemoradiotherapy (CRT, *p* = 0.007) and high pathological stage (*p* = 0.029). Moreover, miR-199b downregulation determined shorter overall (*p* = 0.003) and event-free survival (*p* = 0.005), and was an independent predictor of poor response to preoperative CRT (*p* = 0.004). In conclusion, our findings highlight the clinical impact of miR-199b downregulation predicting poor outcome and pathological response in LARC, and suggest the miR-199b/SET signaling axis as a novel molecular target to prevent the development of resistance to 5-FU treatment.

## 1. Introduction

Colorectal cancer (CRC) is a gastrointestinal cancer with the highest incidence rate and is the fourth leading cause of cancer-related death worldwide [1], with rectal cancer accounting for approximately 30% of all colorectal tumors [2]. The guidelines of the National Comprehensive Cancer Network recommend a multidisciplinary approach consisting of 5-fluorouracil (5-FU)-based preoperative CRT followed by total mesorectal excision (TME) surgery that represents the treatment of choice for locally advanced rectal cancer (LARC) [3,4]. Although 5-FU is more used, the oral 5-FU prodrug capecitabine has recently been defined as a useful alternative option [5]. This therapeutic regimen has improved the management of LARC patients and was established after several studies demonstrating that CRT before TME led to lower local recurrence rates than TME followed by adjuvant CRT or TME alone [6,7]. In addition, there is an increasing interest in a novel “watch and wait” approach where highly selected patients with a complete response after neoadjuvant CRT can escape surgery in order to improve their quality of life [8]. The pathological response after the introduction of preoperative CRT is of high clinical relevance since those patients who achieve a complete pathological response are expected to experience significantly improved long-term survival rates compared to others with residual tumor [9,10]. Unfortunately, distant metastases are developed in more than one-third of patients within 10 years from diagnosis and complete pathological response only occurs in less than 20% of cases [11]. The lack of effective biomarkers established in clinical practice able to predict pathological tumor response before treatment represents a major current limitation in LARC clinical management. Some postsurgical prognostic factors have been proposed [12,13], but only limited data about potential markers of response to preoperative CRT have been reported to date [14,15,16]. Therefore, the identification of patients who have a higher possibility of responding to preoperative CRT would be of high relevance in order to improve patient outcomes, reduce both treatment morbidity and delays in the resection of the primary tumor, and allow potential nonresponders to receive alternative therapeutic strategies.

MicroRNAs (miRs) are small noncoding single-stranded RNAs that post-transcriptionally inhibit specific target genes. MiRs have been largely reported to play key roles in human cancer, and they can act as oncogenes or tumor suppressors depending on their targets [17]. Due to the ability to be easily detected in both tumor tissue and blood, miRs have been proposed as promising markers with clinical impact in rectal cancer [18,19]. Despite the well-known role of miR-199b as a tumor suppressor in human cancer, only a limited number of studies have explored the role of miR-199b in CRC. This miR has been reported to contribute to CRC progression through the regulation of the SIRT1/CREB/KISS1 signaling pathway [20], and circNSD2 was found to target miR-199b in CRC cells thereby leading to DDR1/JAG1 activation and facilitating the development of metastatic disease [21]. SET is an endogenous inhibitor of the tumor suppressor PP2A [22]. SET has been reported to play oncogenic roles and be overexpressed in a large number of tumor types. In fact, its inhibition has been proposed as a novel anticancer strategy [23]. Our group reported that miR-199b directly targets SET leading to the activation of the tumor suppressor PP2A, and affects cell viability and oxaliplatin sensitivity in CRC cells. In addition, miR-199b downregulation was found as a frequent alteration predictive of poor outcome in metastatic CRC [24], and a very recent study has shown that high levels of both tissue and exosomal miR-199a/b could be associated with better response to CRT in LARC [25]. However, its potential clinical and therapeutic significance in this disease remains to be fully clarified.

In this study, we investigate the role of miR-199b determining resistance to 5-fluorouracil treatment and analyze its potential clinical impact in the subgroup of LARC patients. We observed that miR-199b enhances 5-FU antitumor properties in a SET-dependent manner, and that acquired resistance to 5-FU is overcome by ectopic expression of miR-199b, SET silencing or treatment with the SET inhibitor compound FTY720. To evaluate its clinical relevance, we quantified miR-199b in a cohort of 110 LARC patients, observing that miR-199b downregulation is a common alteration that independently predicts poor outcome and pathological response to neoadjuvant chemoradiotherapy.

## 2. Experimental Section

### 2.1. Cell Cultures and Transfection

The human colorectal cancer cell lines SW480 (ATCC CCL-228) and HT-29 (ATCC HTB-38), were purchased from the American Type Culture Collection (ATCC, Manassas, VA, USA). Authentication was performed by the authors in all cases (LGC Standards). Cell lines were maintained in RPMI-1640 (Invitrogen, Carlsbad, CA, USA) with 10% fetal bovine serum (FBS) and were grown at 37 °C in a 5% CO_2_ atmosphere. Media were supplemented with penicillin G (100 U/mL), and streptomycin (0.1 mg/mL). Cells were treated with 5-FU (Calbiochem, San Diego, CA, USA) and FTY720 (Calbiochem) at the indicated concentrations for each experimental condition. For transfection experiments, CRC cells were seeded in 6-well plates and transfected with 10 µL of Lipofectamine 2000 (Life Technologies, Carlsbad, CA, USA) and 2 µg of SET plasmidic vector, 75 nM of SET-specific siRNAs designed and synthesized by Dharmacon RNA Technologies or 20 nM of a miR-199b specific mirVana^TM^ miRNA Mimic and Inhibitor (Ambion, Cambridge, UK). To generate 5-FU-resistant cells, SW480 cells were cultured in the presence of increasing doses of 5-FU (three subculturing doses per concentration), starting at 0.1 µM. In order to assess the evolution of resistance, we determined IC50 after every 5-FU concentration point, by using an MTS assay (Promega, Madison, WI, USA) after 24 h of treatment. The resistance of every 5-FU-resistant clone was defined as the ratio between resistant and parental cells IC50 values.

### 2.2. Patient Samples and Pathologic Response

We retrospectively selected and included in this study a total of 110 consecutive specimens from patients with a histological diagnosis of LARC who were treated with preoperative CRT between 2006 and 2014 at the University Hospital Fundación Jiménez Díaz, (Madrid, Spain). All cases had an accurate preoperative locoregional staging based on a magnetic resonance image (MRI) of the pelvis and/or transrectal ultrasound (TRUS). A full-body computed tomography scan (FBCTS) was carried out in all patients in order to exclude metastatic disease. The patients were treated with chemoradiotherapy regimens based on 5-FU and underwent surgery 6 to 8 weeks after neoadjuvant CRT completion. All participants gave written informed consent for tissue storage and analysis at Fundación Jiménez Díaz biobank, Madrid (Spain). The ethical committee institutional review board of Fundación Jiménez Díaz University Hospital reviewed and approved the project (2018/54).

All tumor specimens derived from the surgical resection were classified according to the College of American Pathologist guidelines for invasive carcinomas (TNM, 7th ed.). Two independent pathologists who were blinded to patient outcome evaluated tumor regression grade according to the modified Ryan classification that categorizes tumors into four levels of response: complete response, moderate response, minimal response and poor response. Complete response score 0 indicates no viable cancer cells; moderate score 1 indicates single cells or little groups of cancer cells; minimal score 2 indicates residual cancer outgrown by fibrosis; and poor response score 3 indicates minimal or no tumor kill with extensive residual cancer. According to clinical guidelines, every regression grade was compared with the primary tumor [26].

### 2.3. Nucleic Acid Isolation

Total RNA was isolated from formalin-fixed paraffin-embedded (FFPE) tumor biopsies, applying RecoverAll Total Nucleic Acid Isolation kit Ambion (Thermo Fisher Scientific, Waltham, MA, USA) following manufacturer’s instructions. Total RNA obtained was quantified using a NanoDrop Spectrophotometer (Thermo Scientific, Waltham, MA, USA).

### 2.4. Quantification of miRNA Expression Levels

Total RNA was isolated using the RecoverAll Total Nucleic Acid Isolation kit (Ambion) according to the manufacturer’s instructions. Samples were reverse transcribed using the TaqMan MicroRNA Reverse Transcription Kit (Applied Biosystems, Foster City, CA, USA) and mature miRNAs were quantified by quantitative real-time reverse transcription polymerase chain reaction (RT-PCR) using TaqMan MicroRNA Assays (Applied Biosystems) specific for miR-199b (miR-199b-5p_000500) and U6B as an internal control. Reactions were carried out using an Applied Biosystems 7500 Sequence Detection System. Conditions: 95 °C for 10 min, followed by 45 cycles of 95 °C for 15 s, and 60 °C for 1 min. Analysis of relative gene expression data was performed using the 2−∆∆CT method [27], where ∆∆CT = (CT, Target Gene-CT, U6B) Tumor-(CT, Target Gene-CT, U6B) Normal Control. As previously described [24], downregulation of miR-199b was considered when the expression in a sample was lower than the mean minus standard deviation (SD) of the patient cohort, corresponding to 0.266 fold change.

### 2.5. Cell Viability Assay

Cell proliferation was measured in triplicate wells by the MTS assay in 96-well plates using the CellTiter 96 Aqueous One Solution Cell Proliferation Assay (Promega), according to the manufacturer’s instructions.

### 2.6. Analysis of Caspase Activation

Quantification of caspase-3/7 activities was performed using the caspase Glo-3/7 assay kit (Promega Corp, Madison, WI, USA). Briefly, 5 × 10^3^ cells were seeded in a black-walled 96-well plate, and the Z-DEVD reagent, the luminogenic caspase-3/7 substrate containing a tetrapeptide Asp–Glu–Val–Asp, was added with a 1:1 ratio of reagent to sample. After 90 min at room temperature, the substrate cleavage by activated caspase-3 and -7, and the intensity of a luminescent signal was measured by a FLUOstar OPTIMA luminometer (BMG Labtech, Cary, NC, USA). Differences in caspase-3/7 activity are expressed as fold change in luminescence.

### 2.7. Western Blot Analysis

Protein extracts were isolated using TRIzol Reagent (Invitrogen) following the manufacturer’s indications, clarified (12,000× *g*, 15 min, 4 °C), denatured and subjected to SDS-PAGE and Western blot. Antibodies used were rabbit polyclonal anti-SET (Abcam, Cambridge, UK) and mouse monoclonal anti-βactin (Sigma, St. Louis, MO, USA). Proteins were detected with the appropriate secondary antibodies conjugated to alkaline phosphatase (Sigma) by chemiluminescence using Tropix CSPD and Tropix Nitro Block II (Applied Biosystems).

### 2.8. Statistical Analysis

Statistical analyses were performed using SPSS 20 for windows (SPSS Inc, Chicago, IL USA). We applied the χ2 test (Fisher exact test) based on the bimodal distribution of data to analyze the correlation between miR-199b downregulation and the clinical and pathological variables.

Overall survival (OS) was defined as the time from the date of diagnosis to the date of death or last follow-up. Event-free survival (EFS) was defined as the time from diagnosis until any primary, local or distant recurrence, death or last follow-up. Kaplan–Meier plots and survival comparisons were performed by means of a log-rank test if the proportional hazard assumption was fulfilled and Breslow otherwise. The Cox proportional hazards model was adjusted taking into consideration significant parameters in the univariate analysis. The cutoff point for miR-199b expression was determined as previously described [24]. This work was carried out in accordance with Reporting Recommendations for Tumor Marker Prognostic Studies (REMARK) guidelines [28]. Data represented for transfection experiments are mean of three independent experiments ± s.d. Statistical comparisons were carried out by two-sided *t*-test analyses; *p* < 0.05 was considered statistically significant.

## 3. Results

### 3.1. MiR-199b Sensitizes to 5-FU Treatment in a SET-Dependent Manner

**Figure 1:**
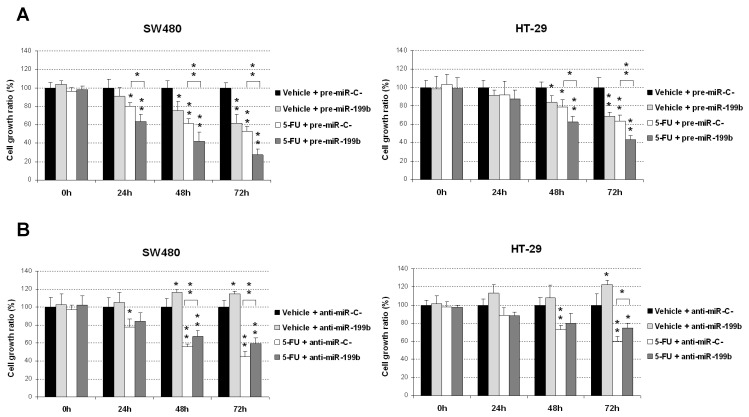
MiR-199b enhances sensibility to 5-fluorouracil (5-FU) treatment. MTS assay showing cell growth in SW480 and HT-29 cells treated with 5-FU (1 μM) and transfected with (**A**) pre-miR-199b or (**B**) anti-miR-199b; * *p* < 0.05; ** *p* < 0.01.

To fully clarify the biological relevance of miR-199b regulating 5-FU efficacy, we first confirmed previous findings reporting that miR-199b overexpression enhances 5-FU antitumor effects. As expected, we observed that miR-199b overexpression significantly enhanced 5-FU-induced antitumor properties in both SW480 and HT-29 cells in comparison with those cells transfected with a negative control miRNA (Figure 1A). As the effects of miR-199b downregulation have not been previously reported we transfected both cell lines with a specific anti-miR-199b to further validate the observations overexpressing miR-199b. Interestingly, we observed a reduction in sensitivity to 5-FU in both cases (Figure 1B). These results confirm that miR-199b is involved in modulating the sensitivity of CRC to 5-FU treatment.

**Figure 2:**
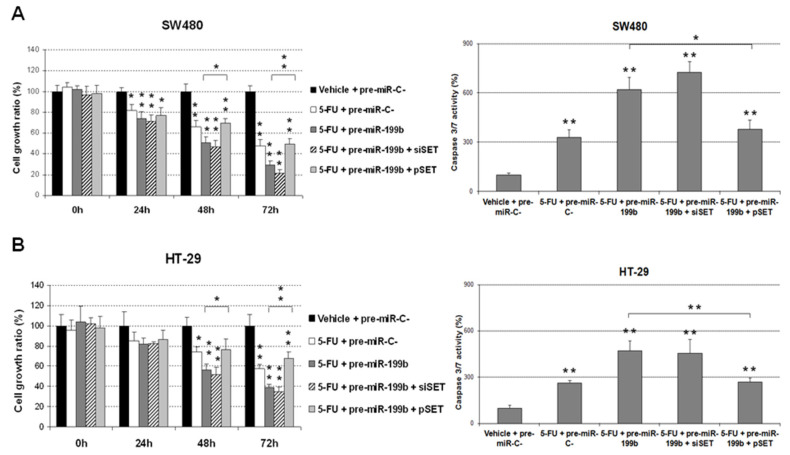
MTS and caspase 3/7 assays showing the effects of an ectopic SET modulation in miR-199b-dependent 5-FU resensitization in (**A**) SW480 and (**B**) HT-29 cells; * *p* < 0.05; ** *p* < 0.01.

As SET is a miR-199b target which has been previously described to play a key role in miR-199b-mediated oxaliplatin resensitization in metastatic CRC, we next analyzed the potential role of SET in the effects observed on 5-FU treatment after modulation of miR-199b expression. Interestingly, we observed that the ectopic expression of SET completely restored the enhanced 5-FU antitumor effects induced by miR-199b overexpression in SW480 cells in both cell growth and caspase-dependent apoptosis. However, when we silenced SET in SW480 cells ectopically expressing miR-199b, we observed that the cell growth reduction after 5-FU treatment was even greater. In concordance, both miR-199b expression and SET silencing markedly enhanced cell apoptosis (Figure 2A). Similar results were found in HT-29 cells (Figure 2B). Altogether, these results would indicate that SET regulation is a key event that mediates miR-199b-induced effects on 5-FU sensitivity.

### 3.2. The miR-199b/SET Axis Emerges as a Novel Target to Overcome 5-FU Resistance

**Figure 3:**
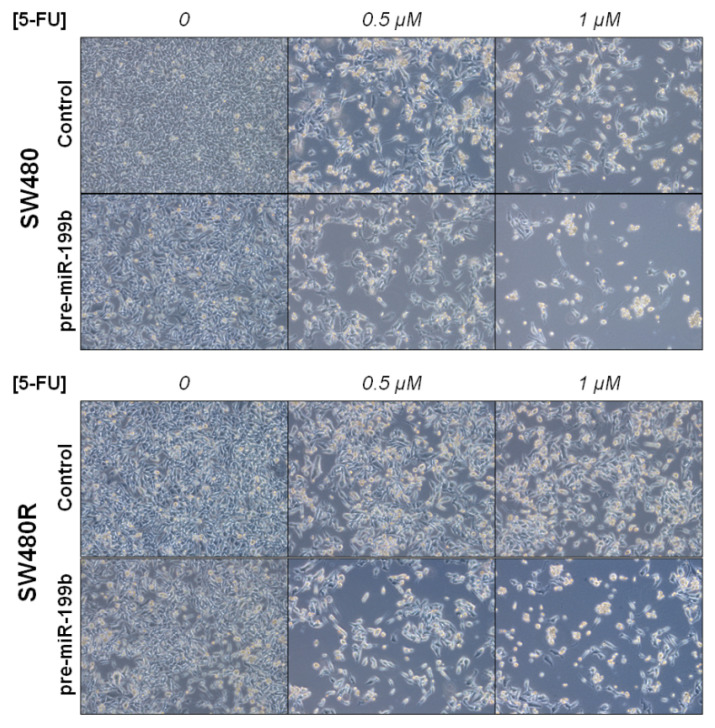
Optical microscope images (magnification ×100) showing miR-199b-induced changes in cell viability after 5-FU treatment in SW480 and SW480R.

To further explore the potential relevance of miR-199b in determining a 5-FU resistance phenotype, we treated SW480 and SW480R cells with different 5-FU concentrations (0.5 and 1 μM) for 72 h and observed that the ectopic expression of miR-199b resensitized SW480R cells to 5-FU at levels similar to SW480 cells (Figure 3).

**Figure 4:**
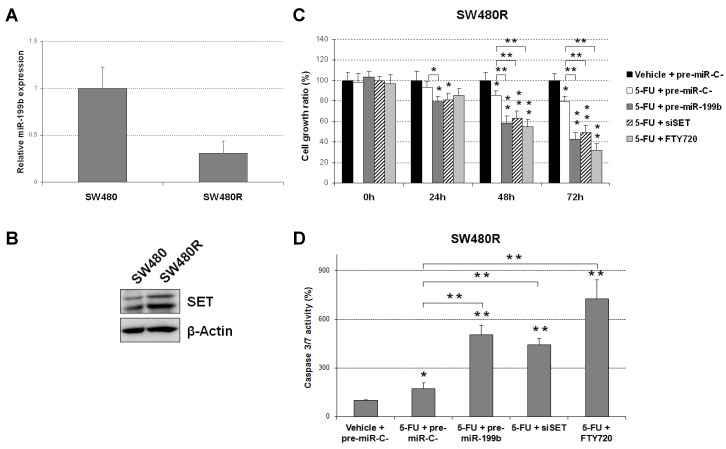
The miR-199b/SET axis is involved in 5-FU resistance. (**A**) Real-time PCR showing relative miR-199b expression in SW480 and SW480R cells; (**B**) Western blot analysis of SET expression in SW480 and SW480R cells; (**C**) MTS assay and (**D**) caspase 3/7 assay showing effects of miR-199b overexpression or SET inhibition in SW480R cells treated with 5-FU (1 μM); * *p* < 0.05; ** *p* < 0.01. Detailed information about western blot can be found in Figure S2.

Next, we analyzed miR-199b levels in SW480 cells with acquired resistance to 5-FU treatment (SW480R), observing an almost three-fold decrease in miR-199b expression in these cells in comparison with parental SW480 (5-FU-sensitive, Figure 4A). In concordance with these results, SW480R cells showed a marked increase in SET expression levels (Figure 4B). In order to investigate whether SET plays a role in determining miR-199b-dependent sensitivity to 5-FU treatment and its potential value as a novel target to overcome 5-FU resistance, we performed an MTS assay in SW480R cells treated with 5-FU. In concordance with our last observations, we found that miR-199b overexpression markedly resensitized SW480R cells to 5-FU. Moreover, we observed that both genomic and pharmacological SET inhibition had similar effects (Figure 4C). To further confirm these observations, caspase activation assays were carried out, observing that miR-199b overexpression and SET inhibition strongly enhanced 5-FU-induced cell apoptosis in SW40R cells (Figure 4D). Two different siRNAs were used for SET silencing and similar effects were observed (Figure S1A). No differences were found between controls including pre-miR-C-, siRNA-C- and DMSO (Figure S1B). Altogether, these results appear to indicate that the miR-199b/SET signaling axis is involved in 5-FU resistance and emerges as a novel druggable target to overcome that resistance using compounds such as FTY720.

### 3.3. Prevalence of miR-199b Downregulation and Its Association with Molecular and Clinical Parameters

To study the prevalence and clinical relevance of miR-199b, we quantified the expression of this miR in a cohort of 110 LARC patients, observing miR-199b downregulation in 26.4% of cases (29 out of 110). Patient characteristics are shown in Table S1. Next, we analyzed the association of low miR-199b levels with clinical and molecular characteristics in 82 LARC patients from our cohort with clinical follow-up data available. MiR-199b downregulation correlated with positive lymph node levels after CRT (*p* = 0.007) and high pathological stage (*p* = 0.029). Moreover, patients with low miR-199b levels had higher tumor size after CRT, however, statistical significance was not achieved (*p* = 0.143). The association between miR-199b expression and molecular and clinical parameters are included in Table 1.

Since we observed differences in both tumor size and lymph node positivity between high and low miR-199b expression, we next analyzed which patient subgroups are different. We observed that those cases with low tumor size (*p* = 0.015) and lack of lymph nodes (*p* = 0.006) significantly associated with miR-199b downregulation (Table S2).

### 3.4. MiR-199b Predicts Pathological Response to Neoadjuvant CRT in Locally Advanced Rectal Cancer

To investigate the potential predictive value of response to preoperative CRT of miR-199b, we grouped our patient cohort into responders and nonresponders. Interestingly, we observed that low miR-199b expression is strongly associated with a lack of response (70.8% vs. 34.5%, *p* = 0.004, Table 2). In concordance with these observations, low miR-199b expression significantly associated with a lack of downgrading when we compared the grade pre and post-CRT in 60 cases with clinical data available (Table S3).

**Figure 5:**
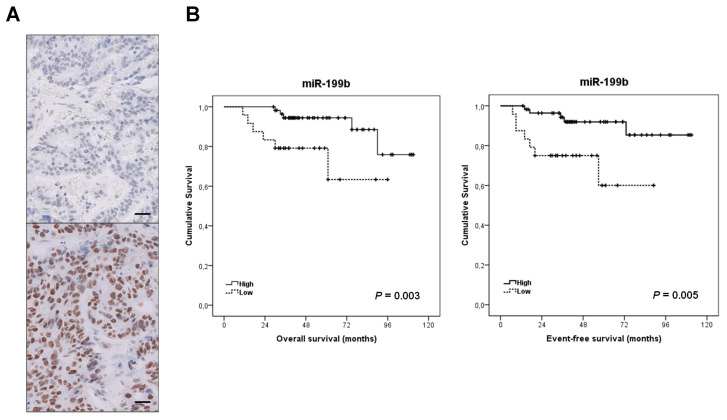
Clinical significance of the miR-199b/SET axis in LARC. (**A**) Immunohistochemical detection of SET in LARC patients showing SET-negative and positive stainings. The line shows 25 µm. Original magnification ×400; (**B**) Kaplan–Meier analyses for miR-199b expression in LARC patients.

Furthermore, we quantified SET by RT-PCR in 76 out of 82 cases with enough material available. We found SET overexpressed in 22.4% of cases and significantly correlated with low miR-199b expression (*p* = 0.004, Table S4) and lack of response to neoadjuvant CRT (*p* = 0.015, Table S5). To further confirm the inverse correlation between SET and miR-199b expression, we analyzed SET by immunohistochemistry in a set of 20 LARC cases, observing SET overexpression associated with those patients with low miR-199b levels (Figure 5A and Table S6).

Notably, multivariate analysis demonstrated that preoperative miR-199b expression is an independent predictor of response to neoadjuvant CRT in our cohort of LARC patients (*p* = 0.004, Table 3).

### 3.5. Clinical Significance of miR-199b Downregulation in Locally Advanced Rectal Cancer

To further investigate the impact of miR-199b in LARC, we next analyzed its potential value predicting clinical outcome. For survival analyses we included those 82 LARC cases with clinical follow-up data available, 49 were male and 33 female, with a median of age of 69 years (range: 46–85). Interestingly, we found that the subgroup of patients with low miR-199b had a substantially shorter OS (59 vs. 94 months, *p* = 0.003) and EFS (65 vs. 102 months, *p* = 0.005, Figure 5B).

Interestingly, multivariate analysis demonstrated that low miR-199b levels are an unfavorable independent factor associated with both OS and EFS in our cohort (Table 4 and Table S7).

## 4. Discussion

There is an urgent need to establish robust markers predictive of response to neoadjuvant CRT in LARC. Although some gene signatures and specific miR profiles have shown potential prognostic value, none of them have been well-validated and incorporated in the clinical routine [15,19]. We previously reported that miR-199b deregulation is a common alteration in metastatic CRC and demonstrated that the PP2A inhibitor SET is a direct target of this miR [24]. PP2A is a key tumor suppressor in human cancer due to its regulatory role in signaling pathways crucial for the tumor cells [29,30]. Several studies have shown the high relevance of PP2A inhibition in CRC [31,32], and the overexpression of its endogenous inhibitor SET [22] as a primary alteration to inactivate PP2A in this disease [33]. Our group reported that SET overexpression induces cell growth and decreases the sensitivity of CRC cells to standard chemotherapeutic agents such as oxaliplatin and 5-FU. As expected with the reported role of SET modulating sensitivity of CRC cells to oxaliplatin and 5-FU [33], miR-199b was found to also increase the effects of both treatments [24]. Considering that preoperative CRT regimens in LARC are based on 5-FU, we hypothesized that this miR could have a potential clinical and therapeutic impact on this disease. Thus, our results in Figure 1A are concordant with previous observations indicating that miR-199b overexpression sensitizes CRC cells to 5-FU treatment [24]. Moreover, we also used a specific anti-miR-199b, observing an expected reduction in the 5-FU antitumor effects in both CRC cell lines tested (Figure 1B) which further confirmed the role of miR-199b modulating sensitivity to this drug. The fact that the observed effects with the anti-miR-199b were of less intensity is probably due to the fact that both SW480 and HT-29 cells have a low basal miR-199b expression. Next, we showed that miR-199b sensitizes to 5-FU in a SET-dependent manner (Figure 2), similarly to the observations previously reported with oxaliplatin [24]. In order to demonstrate the involvement of SET we ectopically overexpressed SET in miR-199b-expressing CRC cells using a plasmidic vector without the 3′UTR region and the specific miR-199b “seed region”, which makes miR-199b unable to inhibit the ectopic SET. In these conditions, SET completely restored the miR-199b-induced effects demonstrating that this miR modulates 5-FU sensitivity at least partially through its target SET. However, we cannot claim other contributing targets in addition to SET and the total reversion could also be due to the high ectopic overexpression levels achieved. In fact, SIRT1 is another miR-199b target which has been shown to contribute to 5-FU resistance in CRC cells [34,35]. In addition, the enhanced effects after FTY720 treatment could be explained by additional antitumor effects to SET inhibition such as CIP2A downregulation [31] or by the fact that miR-199b overexpression does not lead to a total endogenous SET inhibition [36]. In order to demonstrate the potential therapeutic value of the miR-199b/SET axis, we generated an acquired resistant model of CRC cells to 5-FU observing that both miR-199b overexpression and genetic or pharmacologic SET inhibition are able to overcome 5-FU resistance (Figure 4), which suggests that these alterations could serve as novel targets for alternative therapeutic strategies in LARC patients with a lack of response to standard preoperative CRT. In fact, the use of SET antagonists such as FTY720 and OP449 have shown promising effects in SET-overexpressing human tumors and could be useful in LARC patients [37,38]. Previous findings have shown that miR-199b downregulation predicts poor outcome in metastatic CRC. Moreover, our group recently reported that miR-199b downregulation was significantly associated with SET overexpression in a set of 29 CRC patients without metastatic disease. Notably, seven out of 29 cases had SET overexpressed, and three out of those seven SET-overexpressing cases showed a concomitant miR-199b downregulation, which suggests that this alteration is a contributing mechanism to deregulate SET in early-stage CRC [39]. We observed here an inverse correlation between miR-199 and SET expression in LARC. We found low miR-199b levels in 10 out of 17 SET-overexpressing cases, which suggest the existence of additional mechanisms to deregulate SET in LARC. Moreover, both SET and miR-199b determined response to neoadjuvant CRT, highlighting the clinical impact of the miR-199b/SET axis in this disease. Although there is no current data about the potential prognostic value of miR-199b in early-stage CRC, our results showing that low miR-199b levels predict shorter OS and EFS in LARC (Figure 5) together with the fact that SET predicts poor outcome in CRC highlights that miR-199b could be of clinical relevance in CRC without metastatic disease. Moreover, miR-199b downregulation predicted the lack of response to neoadjuvant CRT in our cohort, results that are strengthened by the findings recently reported in 65 LARC tumor samples indicating that high miR-199a/b levels predicted better response to preoperative CRT [25]. Moreover, high exosomal miR-199b expression also correlated with CRT response, which indicates the potential usefulness of this miR for liquid biopsies. This will need to be validated in further randomized, controlled studies before inclusion in clinical protocols.

## 5. Conclusions

In conclusion, miR-199b downregulation is a frequent alteration in LARC that contributes to 5-FU resistance in a SET-dependent manner. Moreover, both miR-199b overexpression and SET inhibition can overcome the 5-FU-resistant phenotype. Our results indicate that low levels of miR-199b independently predicts shorter OS and EFS, and could be used to anticipate poor pathological response to neoadjuvant CRT in LARC patients. Altogether, our findings highlight the potential impact of the miR-199b/SET axis as a novel therapeutic strategy in LARC, which needs to be fully confirmed in forthcoming studies including ex vivo and in vivo models.

## Figures and Tables

**Table 1:** Association between miR-199b and clinical and molecular parameters in 82 locally advanced rectal cancer (LARC) patients.

Parameters	No. Cases	No. miR-199b High (%)		No. miR-199 Low (%)		*p*
**MiR-199b**	82	58	(70.7)	24	(29.3)	
**Age**	82	58		24		0.407
**<70**	42	28	(48.3)	14	(58.3)	
**≥70**	40	30	(51.7)	10	(41.7)	
**ECOG ^1^**	82	58		24		0.370
**0**	52	35	(60.3)	17	(70.8)	
**1–2**	30	23	(39.7)	7	(29.2)	
**Clinical Stage Pre-CRT ^2^**	82	58		24		0.638
**II**	5	4	(6.9)	1	(4.2)	
**III**	77	54	(93.1)	23	(95.8)	
**Grade Pre-CRT**	73	50		23		0.185
**Low**	21	12	(24)	9	(39.1)	
**Moderate-High**	52	38	(76)	14	(60.9)	
**ypT ^3^**	82	58		24		0.143
**0**	11	10	(17.2)	1	(4.2)	
**1**	11	10	(17.2)	1	(4.2)	
**2**	26	16	(27.6)	10	(41.6)	
**3**	27	19	(32.8)	8	(33.4)	
**4**	4	2	(3.4)	2	(8.3)	
**x**	3	1	(1.8)	2	(8.3)	
**ypN ^4^**	82	58		24		**0.007**
**N0**	64	50	(86.2)	14	(58.3)	
**N1**	14	5	(8.6)	9	(37.5)	
**N2**	4	3	(5.2)	1	(4.2)	
**Pathological Stage**	82	58		24		**0.029**
**yp0**	11	10	(17.2)	1	(4.2)	
**ypI**	32	25	(43.1)	7	(29.2)	
**ypII**	21	15	(25.9)	6	(25)	
**ypIII**	18	8	(13.8)	10	(41.6)	

^1^ ECOG = Eastern Cooperative Oncology Group; ^2^ CRT = chemoradiotherapy; ^3^ ypT = tumor size after CRT; ^4^ ypN = pathological lymph node after CRT.

**Table 2:** Association between miR-199b expression levels and pathological response to neoadjuvant CRT in LARC patients.

Responders vs. Nonresponders						
MiR-199b Expression	No. Cases	MiR-199b Low (%)		MiR-199b High (%)		*p*
**Response**	82	24		58		0.004
**Nonresponse ^1^**	37	17	(70.8)	20	(34.5)	
**Response ^2^**	45	7	(29.2)	38	(65.5)	

^1^ Nonresponse: poor or minimal pathological response; ^2^ Response: moderate or complete pathological response.

**Table 3:** Univariate and multivariate logistic analyses for pathological response in 82 LARC patients.

Response ^1^ vs. Nonresponse ^2^	Univariate Analysis				Multivariate Analysis			
	OR ^4^	95% CI ^3^		*p*	HR	95% CI		*p*
		Lower	Upper			Lower	Upper	
Age				0.192				0.095
<70	1.000				1.000			
≥70	1.796	0.746 to 4.327			2.275	0.868 to 5.968		
Gender				0.193				-
Male	1.000							
Female	0.549	0.222 to 1.354			-	-		
Clinical Stage				0.551				-
II	1.000							
III	1.307	0.543 to 3.144			-	-		
Grade Pre-CRT ^5^				0.605				-
Low	1.000							
Moderate-High	1.185	0.623 to 2.252			-	-		
ECOG ^6^				0.501				-
0	1.000							
1–2	1.364	0.553 to 3.363			-	-		
MiR-199b				0.004				0.002
High	1.000				1.000			
Low	4.614	1.642 to 11.969			5.332	1.813 to 13.679		

^1^ Response: moderate or complete pathological response; ^2^ Nonresponse: poor or minimal pathological response; ^3^ CI: confidence interval; ^4^ OR: odds ratio; ^5^ CRT: chemoradiotherapy; ^6^ ECOG: Eastern Cooperative Oncology Group.

**Table 4:** Univariate and multivariate Cox analyses in the cohort of 82 LARC patients.

Parameters	Univariate OS ^1^ Analysis				Multivariate OS Cox Analysis			
	HR ^3^	95% CI ^2^		*p*	HR	95% CI		*p*
		Lower	Upper			Lower	Upper	
Age				0.261				0.184
<70	1.000				1.000			
≥70	1.914	0.617 to 5.937			2.249	0.680 to 7.436		
Pathological Stage				0.206				-
0–I	1.000							
II–III	2.063	0.672 to 6.632			-	-		
ypT ^4^				0.204				-
0–2	1.000							
3–4	1.274	0.877 to 1.850			-	-		
ypN ^5^				0.048				0.108
N−	1.000				1.000			
N+	3.336	1.011 to 11.005			2.243	0.861 to 4.544		
ECOG ^6^				0.434				-
0	1.000							
1–2	1.549	0.518 to 4.632			-	-		
MiR-199b				0.014				0.040
High	1.000				1.000			
Low	4.036	1.328 to 12.261			3.478	1.059 to 11.425		

^1^ OS: overall survival; ^2^ CI: confidence interval; ^3^ HR: hazard ratio; ^4^ ypT: tumor size after chemoradiotherapy (CRT); ^5^ ypN: pathological lymph node after CRT; ^6^ ECOG: Eastern Cooperative Oncology Group.

## Supplementary Materials

The following are available online at https://www.mdpi.com/2072-6694/12/6/1655/s1, Figure S1: Controls for MTS and caspase 3/7 assays, Figure S2: Detailed information about SET analysis by western blot, Table S1: Association between mir-199b and clinical and molecular parameters in 110 LARC patients, Table S2: Association between mir-199b and tumor size/lymph node positivity in 82 LARC patients, Table S3: Association between miR-199b expression levels and downgrading in 60 LARC patients, Table S4: Association between SET and miR-199b expression in LARC patients, Table S5: Association between SET and pathological response to neoadjuvant CRT in LARC patients, Table S6: Association between miR-199b and immunohistochemical SET expression in LARC patients, Table S7: Univariate and multivariate Cox analyses for EFS in the cohort of 82 LARC patients.
