# Supplementary material for: MicroRNA-199b Downregulation Confers Resistance to 5-Fluorouracil Treatment and Predicts Poor Outcome and Response to Neoadjuvant Chemoradiotherapy in Locally Advanced Rectal Cancer Patients
Source: Cancers (Basel). 2020 Jun 22;12(6):1655. doi: 10.3390/cancers12061655 (PMC7352382; doi:10.3390/cancers12061655)
Supplement: Supplementary file 1 [file cancers-12-01655-s001.pdf]

Supplementary Materials

MicroRNA-199b downregulation confers resistance to 5-fluorouracil treatment and predicts poor outcome and response to neoadjuvant chemoradiotherapy in locally advanced rectal cancer patients

Ion Cristóbal, Jaime Rubio, Andrea Santos, Blanca Torrejón, Cristina Caramés, Laura Imedio, Sofía Mariblanca, Melani Luque, Marta Sanz-Alvarez, Sandra Zazo, Juan Madoz-Gúrpide, Federico Rojo and Jesús García-Foncillas

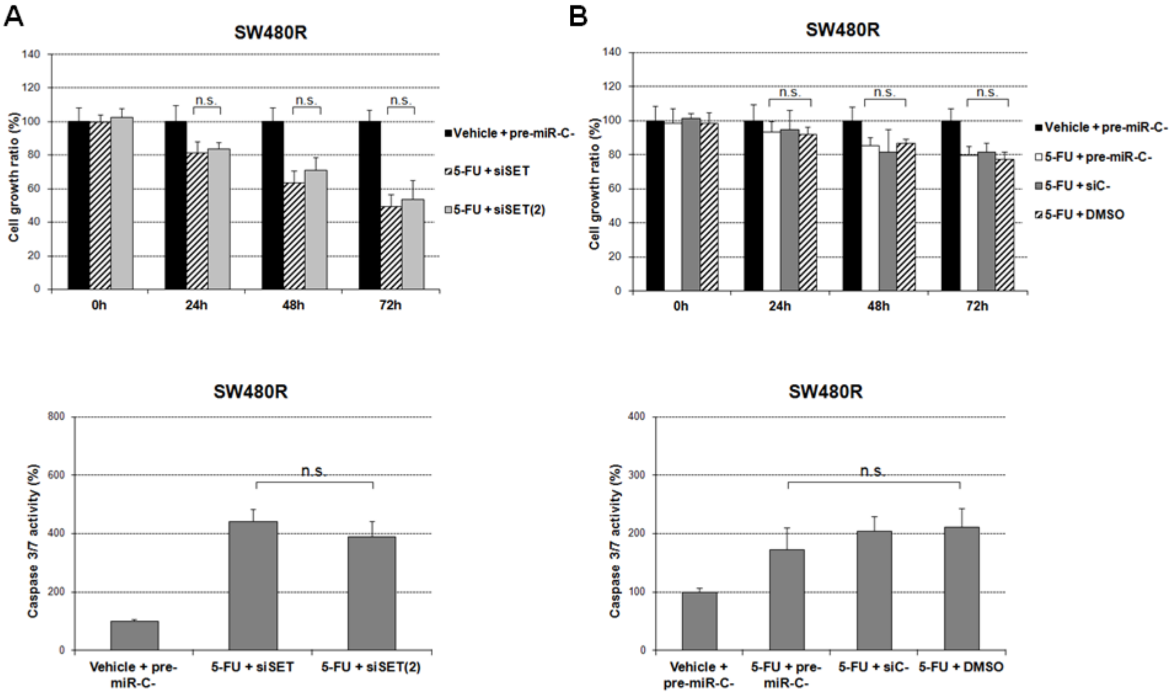

Figure S1. Controls for MTS and caspase 3/7 assays using (**A**) two specific SET siRNAs and (**B**) negative controls for miR-199b, siSET and FTY720.

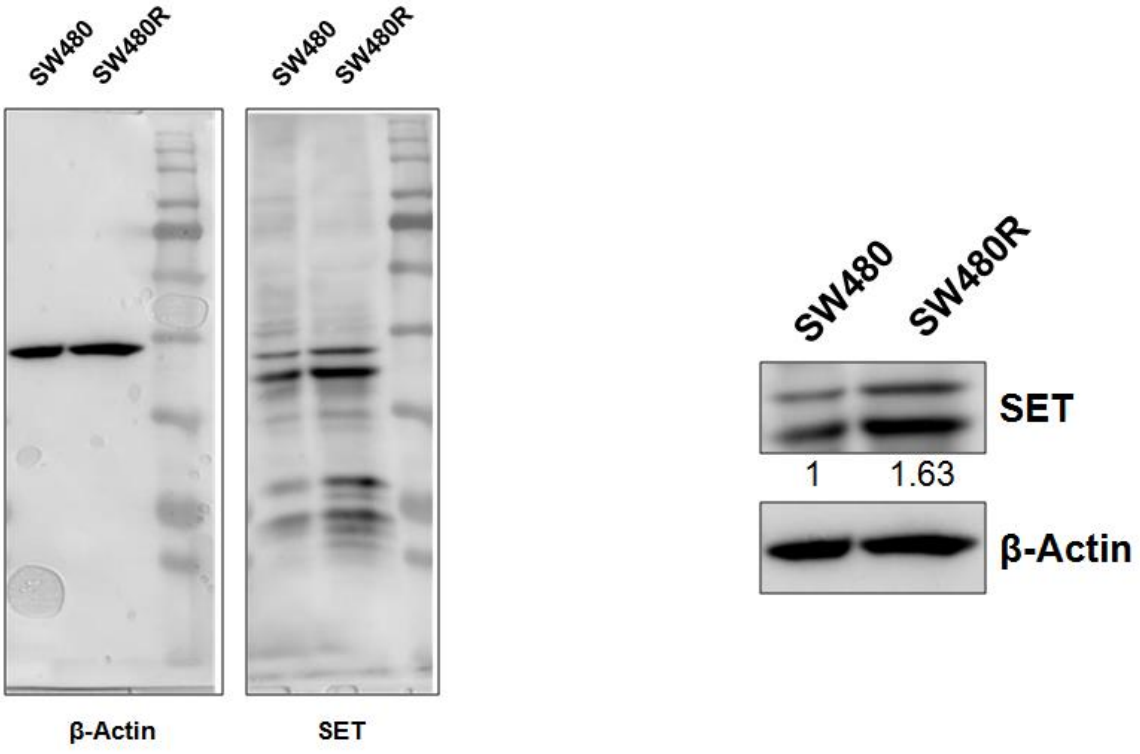

Figure S2. Detailed information about SET analysis by western blot.

Table S1. Association between mir-199b and clinical and molecular parameters in 110 LARC patients.

Parameters	No. Cases	No. miR-199b High (%)		No. miR-199 Low (%)		p
MiR-199b	110	81	(73.6)	29	(26.4)	
Age	110	81		29		0.919
< 70	56	41	(73.2)	15	(26.8)	
≥ 70	54	40	(74.1)	14	(25.9)	
ECOG	110	81		29		0.300
0	67	47	(70.1)	20	(29.9)	
1–2	43	34	(79.1)	9	(20.9)	
Stage	110	81		29		0.547
I–II	15	12	(80)	3	(20)	
III	95	69	(72.6)	26	(27.4)	

Table S2. Association between mir-199b and tumor size/lymph node positivity in 82 LARC patients.

Parameters	No. Cases	No. miR-199b High (%)		No. miR-199 Low (%)		p
ypT	82	58		24		0.015
0–1	22	20	(34.5)	2	(8.3)	
> 1	60	38	(65.5)	22	(91.7)	
ypN	82	58		24		0.006
N0	64	50	(86.2)	14	(58.3)	
N+	18	8	(13.8)	10	(41.7)	

Table S3. Association between miR-199b expression levels and downgrading in 60 LARC patients.

MiR-199b Expression	No. Cases	MiR-199b Low (%)		MiR-199b High (%)		p
Downgrading	60	21		39		0.004
No	40	19	(90.5)	21	(53.8)	
Yes	20	2	(9.5)	18	(46.2)	

Table S4. Association between miR-199b and *SET* expression levels in LARC patients.

Parameters	No. Cases	MiR-199b Low (%)		MiR-199b High (%)		*p*
SET	76	23		53		0.004
Low	59	13	(56.5)	46	(86.8)	
High	17	10	(43.5)	7	(13.2)	

Table S5. Association between *SET* expression levels and pathological response to neoadjuvant CRT in LARC patients.

Responders vs. Non-Responders						
***SET* Expression**	**No. Cases**	***SET* Low (%)**		***SET* High (%)**		***p***
Response	76	59		17		0.015
Non-Response[1]	34	22	(37.3)	12	(70.6)	
Response[2]	42	37	(62.7)	5	(29.4)	

[1]Non-Response: poor or minimal pathological response; [2]Response: moderate or complete pathological response.

Table S6. Association between miR-199b and immunohistochemical SET expression in LARC patients.

Parameters	No. Cases	MiR-199b Low (%)		MiR-199b High (%)		*p*
SET	20	4		16		0.028
Low	17	2	(50)	15	(93.7)	
High	3	2	(50)	1	(6.3)	

Table S7. Univariate and multivariate Cox analyses in the cohort of 82 LARC patients.

Parameters	Univariate EFS[1] Analysis			Multivariate EFS Cox Analysis		
	HR[3]	95% CI[2] Lower Upper	*p*	HR	95% CI Lower Upper	*p*
Age			0.427			0.384
< 70	1.000			1.000		
≥ 70	1.573	0.514 to 4.817		1.686	0.520 to 5.468	
Gender			0.966			-
Male	1.000					
Female	0.976	0.318 to 2.997		-	-	
Pathological Stage			0.211			-
0–I	1.000					
II–III	2.050	0.666 to 6.313		-	-	
ypT[4]			0.149			-
0–2	1.000					
3–4	1.318	0.906 to 1.916		-	-	
ypN[5]			0.040			0.076
N-	1.000			1.000		
N+	3.522	1.057 to 11.738		2.045	0.929 to 4.504	
ECOG[6]			0.341			-
0	1.000					
1–2	1.702	0.570 to 5.081		-	-	
MiR-199b			0.020			0.042
High	1.000			1.000		
Low	3.744	1.226 to 11.428		3.360	1.042 to 10.831	

[1]EFS: event-free survival; [2]CI: confidence interval; [3]HR: Hazard ratio; [4]ypT: tumor size after chemoradiotherapy (CRT); [5]ypN: pathological lymph node after CRT; [6]ECOG: Eastern Cooperative Oncology Group.

© 2020 by the authors. Licensee MDPI, Basel, Switzerland. This article is an open access article distributed under the terms and conditions of the Creative Commons Attribution (CC BY) license (http://creativecommons.org/licenses/by/4.0/).
